# Infectious Diseases of Our Farm Animals1Presented at the sixth annual meeting of the National Live-stock Association, Kansas City, Missouri, January 15, 1903.

**Published:** 1903-04

**Authors:** W. H. Dalrymple

**Affiliations:** Louisiana State University and A. and M. College, Baton Rouge, La.


### THE JOURNAL

OF

### COMPARATIVE MEDICINE AND VETERINARY ARCHIVES.

VOL. XXIV. *APRIL, 1903.* No. 4.

### INFECTIOUS DISEASES OF OUR FARM ANIMALS.[1]

BY W. H. DALRYMPLE, M.R.C.V.S.,

LOUISIANA STATE UNIVERSITY AND A. AND M. COLLEGE, BATON ROUGE, LA.

MR. PRESIDENT: When we consider for one moment that the farm animals of the United States represent, in money value, close upon three billions of dollars, and that a single disease of the zymotic class may destroy some millions of dollars worth of live-stock property in the course of a single year, the more intelligent discussion of the subject allotted to me, including effective measures for the control and extermination of the infectious diseases of our farm animals, is one of such momentous importance to the general well-being of the country that it might with benefit have been entrusted to much abler hands than mine. True, there are some of these plagues that we have had to deal with somewhat extensively during the past ten years or so, but we scarcely look upon that limited experience as sufficient to render one altogether *au fait* in matters connected with the entire group, which the title of our paper would seem to comprehend, so that we will have to treat these maladies in a rather cursory manner, and probably summon to our aid the experiences of others to help us out. Nor could it be expected that within the limits of an ordinary paper, and that, too, of a somewhat popular character, and before an audience presumed to be composed of an admixture of both scientific and practical men, so called, the long list of infectious diseases could be discussed in anything like an exhaustive manner as to thoroughness and detail.

[1] Presented at the sixth annual meeting of the National Live-stock Association, Kansas City, Missouri, January 15, 1903.

Gentlemen, the more that this colossal subject occupies our attention and our time, the more do we become convinced of its tremendous import and of the urgent necessity for co-operation—in fact, united effort—if we are to obtain the maximum of success in dealing with those destructive agencies which annually cost the community, the State, and the country so much money.

Perhaps the most powerful factor that has to be reckoned with today, as has been the case in the past (in preventing greater progress with the bacterial diseases of our live-stock), is an insufficiently educated public opinion, although writings on health are among the oldest in the world, and the subject has engaged the attention of the profoundest thinkers and the most renowned leaders of men.

The immortal Parkes, the founder of modern hygiene, tells us that "if we had a perfect knowledge of the laws of life, and could practically apply this knowledge in a perfect system of hygienic rules, disease would be impossible. That such a perfect knowledge of these laws is not likely to be obtained, or rather, if obtained, is not likely to be acted upon, we have no reasonable doubt. The value of health will never be generally appreciated, and the serious losses occasioned by disease seem only too readily effaced from the public mind."

It is the province of hygiene to seek out and determine the causes of disease and to formulate rules for their prevention and removal. It is a matter of impossibility, however, for the layman to make a study and familiarize himself with all of the agencies at work in the production of disease. This implies a lifework, and, in the investigation of the numerous contagious diseases, a knowledge of the life-histories of the various pathogenic or disease-producing organisms, such as their identification, requirements for existence, methods of reproduction, modes of spread, the germicidal agents by which they may be effectually destroyed, the varying degrees of tenacity of life with which they are endowed, etc. These are matters which have to be left to the scientific investigator in the great field of bacteriology, to whom we are so much indebted for the great advancement made in recent years in the control of the many contagious and fatal diseases which have for centuries menaced the lives, both of mankind and animals, throughout the civilized world. But it is possible, and a matter of the greatest importance, that our stock-owning public should at least acquaint themselves with the more practical points demonstrated and the accurate conclusions arrived at by the scientist in connection with this class of diseases which may periodically occur to decimate our herds and flocks, occasion pecuniary loss, and often financial ruin to unfortunate owners and to the country.

It may not, of course, be necessary that the stock-owner should know the minute differential characteristics which distinguish the germ of anthrax from that of tuberculosis under the microscope, nor their differences in measurement by microns; but he ought to know, and it would be to his advantage to know, and that as thoroughly as possible, the more intelligent sanitary methods to employ in order to place a check upon and limit the spread of those dangerous enemies to the health of his animals—the infectious diseases.

Wherever there exists a lack of such knowledge in this country, there is, I think, absolutely no good reason for it. Reliable literature bearing upon the subject abounds, and can be obtained free, or at a minimum of cost, in the form of publications of the National Department of Agriculture, bulletins of our agricultural experiment stations, and in many of the most reputable of our agricultural journals throughout the country. But, notwithstanding these sources of information, it seems frequently to be the case that when a calamity in the guise of a decimating disease attacks our live-stock we are found in supreme ignorance of the proper methods of attack and defence; we throw aside our better judgment and open wide our doors to the inroads of the charlatan or the wily fakir, whose opportunity is our adversity. In a section of the country where live-stock sanitary legislation is not as yet just as stringent as it might be it has been my personal observation that there are certain classes of individuals who are, during an outbreak of contagious diseases, extremely dangerous, to say the least. One of these may be said to be the vendor of proprietary medicines, who, through ignorance of the true nature of the disease, but merely on the say-so of someone who has administered or applied his nostrum, advertises his drug, or condition powder, or stock-food, as a "sure cure," and to which the uninformed and unsuspecting stock-owner "catches on."

Another is the "would-be benefactor," who has been similarly deceived into the belief that he has "hit" upon a specific which has "never failed," recommends it to his neighbor in adversity; and a third is the professional fakir, who neither knows nor cares what the disease may be so long as he can "rake in the shekels" from his credulous and confidence-reposing victim. The danger here lies, not in any special harm that might accrue from the remedy (?) *per se*, but in the liability of the disease to spread through absence of any virtue in these so-called "specifics"—so far as the malady itself is concerned—and the total neglect of any sanitary measures, which in reality are the only rational means by which to successfully combat the ravages of zymotic diseases. Education or accurate information along such lines only will release our stock-owners from the thraldom of such ignorance and empiricism. But it will do more. It will tend to rob our contagious maladies of the terror and dread they have for so long held in the minds of the people. And with such information obtained the errors of the past would be relegated to the archives of a bygone age, and the light of reason and intelligence beam forth to obliterate the dark doings of empiricism, superstition, and doubt which have held such an extended reign.

There is another point which I think will bear passing allusion. There seem to be not a few who still harbor extremely grave doubts, or, at all events, have very cloudy ideas relative to the existence of those minute and lowly forms of life which we are pleased to designate germs, bacteria, micro-organisms, etc. True, their existence would be more convincing to many of us were they large enough to be seen with the naked eye hovering around our horse and cow-stables, hog-lots, etc., and if they could be exterminated by means of an organized round-up and a regular germ-killing time. But their microscopic dimensions and their insidious methods of conveyance are by no means proof of their non-existence; and although their parasitic mode of life often results so fatally to ourselves and our animals, they are not the outcome of spontaneous growth, but are a part of the universe of living things just as much as those claiming a higher position in the scale of creation. It is very fortunate, however, that although bacterial forces of life are very numerous, and the majority of them of economic value in this progressive world of ours, the disease-producing forms are relatively few. Nevertheless, when some of the more dangerous of the latter are introduced into new or previously uncontaminated territory, and for want of the proper measures being taken to prevent their multiplication and spread, their ravages frequently become so serious as to interfere with both national and international commerce in live-stock, as is at present the case in the New England States. Contagion or infection, then, is nothing more or less than the presence of these lowly forms of life—that is, the disease-producing class we call bacteria, although it is a fact that those responsible for the production of some of our contagious diseases have not as yet been identified. This, however, is no doubt due to their extremely minute dimensions and to the fact that up to the present time no instruments of sufficient high magnifying power have been produced to discern them. The sole aim then of the sanitarian, in whatever capacity he may be, whether connected with local health boards, State live-stock sanitary commissions, or the National Bureau of Animal Industry, is, by the employment of the most approved and effective means within his power, to destroy these pathogenic bacteria and thereby prevent their propagation and distribution. This, of course, requires training and accurate information on the part of the expert; but there is nothing chimerical about his methods; he is simply putting into practice the knowledge he has acquired from patient investigation and careful observation. The infected area, however, is often permitted to extend for want of proper appreciation of the importance of sanitary measures on the part of stock-owners, that an epizoötic of disease may possibly occur before the authorities are cognizant of its existence. On the other hand, if more accurate information regarding the laws of animal hygiene—which include sanitation—was acquired by owners of live-stock, and by their hearty co-operation with those having authority to look after contagious diseases, many an outbreak which might otherwise assume the proportions of an extensive epizoötic would be "nipped in the bud," and great loss (through destruction and death of affected animals) thereby averted; and besides, the necessity for an embargo being placed on our foreign and domestic trade in cattle in the infected territory be done away with. A stock-owning public, then, able through more accurate information acquired, and willing to lend their united effort to assist those whose duty it is to labor in this important field of veterinary sanitary science, would, in my humble judgment, be the most potent agency for good in the prevention, control, and eradication of those infectious animal plagues that periodically affect our live-stock and carry such loss and devastation in their wake.

Of "The Four Bovine Scourges," viz., rinderpest, contagious pleuropneumonia, foot-and-mouth disease, and tuberculosis, which the late Prof. Thomas Walley, of Edinburgh, compiled in a valuable volume bearing the above title, we are thankful to be able to say that tuberculosis and foot-and-mouth disease only have any existence at all in this country at the present time; and, although the former is much more widespread than we would wish to see it, the United States was evidently free from the latter disease, with one or two minor exceptions, until quite recently, and there is every reason to hope that through the eternal vigilance of our national and other authorities who have the matter in charge, it will be confined to its present temporary habitat, and before many weeks effectually exterminated from our shores. There could be no greater proof, we think, of the value of a thoroughly organized sanitary system in any country than the accomplishment of the monumental task of completely eradicating contagious pleuropneumonia by the indefatigable efforts of the officers of the National Bureau of Animal Industry, that most important branch of the United States Department of Agriculture. It is almost impossible, in fact, to realize the importance of such an achievement to the country, or to endeavor to make a computation of the value of such a result in dollars and cents. True, Great Britain, for example, which is the largest importer of our cattle, still refuses to land our store animals as an extreme precaution, and which seems to us as somewhat unreasonable, considering that we have for quite a considerable length of time been able to show a clean bill of health so far as this disease is concerned. In so far, then, as contagious pleuropneumonia is concerned, I think we may pass it by, with our sincere gratitude to the government authorities for having so effectually, and, we trust, permanently, stamped this menacing disease out of the country.

Rinderpest or cattle-plague may also, I think, be dealt with in a similar summary manner, as we have never suffered from its ravages in this country, and it is to be hoped we never will, although our new possession—the Philippines—has experienced to some extent its destructive effects.

The "great white plague," as tuberculosis is sometimes referred to by the medical world, is a disease about which so much could be said that it would be impossible to give to it the amount of time and space it merits in a paper of this character. We believe, however, that with that concert of intelligent and unselfish action, previously referred to, on the part of our stock-owners and experts, with neither forces having any personal or selfish ends to subserve, and with the means now being taken by the United States Department of Agriculture to prevent the introduction of tuberculous animals from abroad, the time need not be far distant when this disease also may be placed in the category of those that are known in name only and not in reality in connection with our farm animals on this side of the Atlantic.

The last of the "Four Bovine Scourges," so called by Professor Walley, is the one which has been recently causing so much alarm all over the Union, namely, the foot-and-mouth disease, and, as it is no doubt uppermost in the minds of stockmen at the present moment, we will devote a little more space for its consideration in a general way. This disease, also known by the synonyms: epizoötic aphtha, aphthous fever, eczema epizoötica, vesicular epizoötic, murrain, etc., is one the contagious nature of which has been known for quite a considerable length of time, and seems to have been accurately described in the eighteenth century. According to German authorities we find that the disease existed in Southern Germany, in Switzerland, and in Italy from 1809 to 1812, and again from 1819 to 1823. Prof. George T. Brown, of London, states that in 1839 it was introduced into England, where, since that date there have been ten great epizoötics, which number, however, has been added to since Professor Brown made this statement. From 1840 to 1860 it extended throughout all Europe. In 1871 it affected 700,000 animals in France, and nearly the same number in England, 1400 dying, or 1 per cent. In 1883 in Great Britain they had some half a million aphthous fever cases, 60,000 in Prussia, as many in Austria and Italy, and more than 100,000 in Bavaria, including cattle, sheep and hogs. Although many more similar records might be cited from European countries, the above are, I should think, sufficient for the purpose of showing the infectiousness of this disease and the rapidity with which it can spread. Fortunately for us, however, it is largely restricted to parts of Continental Europe, especially the eastern portion, and its introduction into the United States has been hitherto prevented by the strict enforcement of our quarantine measures, with the exception of a few isolated cases between 1870 and 1889, and, until the recent outbreak, the origin of which appears, as far as I am aware, somewhat hypothetically. The specific cause of foot-and-mouth disease has not been definitely ascertained, or, we should rather say, clearly demonstrated. It is the general belief, however, among the ablest authorities that it is a specific infection, and that each outbreak must originate in some previous case or cases, and that the infection may be transmitted directly or indirectly to healthy animals. The virulent material is contained chiefly in the vesicular eruptions in the mouth and on the feet, the discharge from either being the source of its wide distribution. In infected stables and manure it is said to preserve its activity for many months, which may account in a measure for recovered animals remaining contaminated for a long time; and it is considered that the propagation of outbreaks of this malady occur especially along lines of travel and of transit.

The chief factors of mediate or indirect contagion are enumerated as follows: wagons, stables for transient animals, markets, pastures, and common troughs, dealers and persons who have charge of animals, bulls, feed, litter, manure, etc. Herds of swine in transit have been shown to be particularly dangerous. In the same stable transmission may also occur directly from sick to healthy animals, as by licking, or by milkers. Young suckling animals become infected by the ingestion of milk from diseased mothers. And the sick of one species of animals may contaminate healthy members of other susceptible species.

Among the domestic animals it is most commonly observed in cattle, hogs, sheep, and goats, although the horse, dog, cat, and even poultry may be affected by it, and it may attack the human subject as well. The period of incubation, or the time that elapses between that of infection and the manifestation of the symptoms varies, according to Williams, of Edinburgh, from twenty-four hours to three or four days, the average given by German authorities being three to five days. It runs its course in the animal, if uncomplicated by other conditions, in from eight to twenty days.

Without going over the entire train of symptoms that might be patent to the eye of the trained clinical observer, we may state that the more prominent and more easily recognizable evidences to the stockman are: elevation of temperature and indications of febrile disturbance, uneasiness, and, if the thermometer be used, it would be found that the temperature might reach, but rarely exceed, 104° F., inappetence and cessation of rumination, or chewing the cud, the mucous membrane or lining of the mouth becomes red in appearance, there is frequently smacking of the lips, and if the latter are closed for a time the accumulated saliva escapes in quantity as soon as the mouth is again opened. After the initial stage of the fever (about two or three days' duration) the eruption of vesicles or blisters —of different sizes—appears on the mucous membrane of the gums, inner surface of the lips, inside of the cheeks, on the tongue, and on the roof of the mouth. In a short time the delicate structures enclosing the vesicles are separated and thrown off in more or less rounded patches, leaving raw surfaces, which may, however, be again covered, or may be changed into ulcers. Owing to this painful condition saliva accumulates in large quantities and falls from the mouth in long threads or strings. The symptoms in the feet generally follow those of the mouth, although they may also appear at the same time. The skin around the top of the hoof, and especially in the cleft between the claws and at the heels, becomes red, hot, swollen, and painful. This may be observed in one or in several of the feet simultaneously. Within twenty-four to forty-eight hours small blisters begin to form, varying in size, and containing in the beginning a yellowish, limpid fluid, which later becomes turbid and viscid. The animal is lame, both motion and locomotion being extremely painful, so that it is inclined to remain standing or lying down the greater part of the time. After the blisters have ruptured, scars usually form in from eight to twenty days, provided no complications have arisen in the interim. The udder sometimes becomes affected, the teats more particularly being the seat of the eruption. The vesicles in this situation are frequently broken in the operation of milking, and converted into raw, reddened, tender patches, denuded of epidermis or top layer of the skin.

In the hog the disease is said to first appear in the feet; less frequently in the mouth. And in the sheep and goat the condition is usually confined to the claws, and the course of the disease is said to be slower in these animals than in the ox and the hog.

Although, as may be inferred from some of the statistics given, this malady is extremely infectious, causing considerable inconvenience and pecuniary loss, both to the individual and to the country. It has the redeeming feature, so to speak, of not being very fatal in its results, which are given at about from 1 to 4 per cent., except in the case of very young animals.

The treatment from a national standpoint is, of course, preventive; or, in other words, has to be conducted along purely sanitary lines, and as it is a disease of both national and international significance, this comes within the jurisdiction of the authorities at Washington. Still, by the possession of a working knowledge of such a disease, if only to the point of arousing suspicion, and an acquaintance with the elementary principles of sanitary science and how to practically apply them, the stock-owner could often render valuable aid in limiting the disease to its primary focus, and thereby assist in checking its further spread.

There are quite a number of other diseases that would naturally come within the scope of my paper, but which for the lack of time I will of necessity have to pass by. Before drawing to a close, however, I would like to say a few words in connection with anthrax, a disease with which I have had more or less personal experience within the past few years, and which is considered to be one of the most dangerous and fatal, to both mankind and the lower animals, known to medical science.

Anthrax, or as the French element in the Southern part of the country term it, charbon, also belongs to the specific fevers, and is caused by a germ, the bacterium anthracis, which is found in great numbers in the blood of animals that have succumbed to the disease. It would appear that of later years this disease has become more widely distributed over the country, occasioning considerable loss; but the locality which seems to furnish the most suitable conditions for the propagation of the infective germ is along the Lower Mississippi Valley, where it has periodically in past years played great havoc among animals of every breed and variety, man himself not being excepted. Just when this disease was first introduced into the United States it is impossible to say, but that it has existed for many years in the southern section of the country, and especially along the coast line of the Gulf of Mexico, there can be no reasonable doubt, as from personal investigation we have found that it has been known to exist at least as far back as the recollection of the oldest living inhabitants of that section could carry them, but for how much longer it would be impossible to conjecture. In the early days, and even up to ten or twelve years ago, the true nature of anthrax was as a sealed book to the majority of the inhabitants of the stricken districts, and necessarily the importance of fighting infection, rather than attempting to treat the victims of the disease, did not come up for consideration. Sanitary measures were, therefore, altogether neglected, and, in consequence, the contagion was permitted to spread broadcast by the various agencies capable of disseminating it. The chief source of infection in anthrax is the dead animal that has not received the requisite sanitary care in its disposal. The blood of the carcass just after death contains a teeming mass of living infective organisms, and any agent that comes in contact with the blood and is able to convey it is capable of disseminating the virus wherever it may go. Among these may be mentioned surface water, washing the infected blood into streams or creeks and contaminating their banks, which are frequently grazed, or meadows during seasons of overflow; or in the case of refuse water from tanneries where infected hides have been handled, getting to and infecting running streams; foodstuffs that had been raised on infected lands are frequently soiled with the spores of anthrax, and may convey the disease to distant parts. With us in the Lower Mississippi Valley the carrion birds as well as carnivorous and omnivorous animals running at large are often responsible for its distribution after feeding upon the tissues of the diseased cadaver. The numerous varieties of blood-sucking insects, as horse-flies, very frequently transmit the infection; in fact, these flies are, perhaps, the most responsible agents in the southern country in spreading the disease in its external form after feeding upon the germ-laden blood of the anthrax carcass, which they will do so long as this fluid remains in a warm condition. Pastures that have become infected transmit the disease through the grazing. People frequently contract external anthrax (malignant pustule) through inoculation of wounds on their hands during the process of handling or skinning the dead animals. The disease is quite frequently imported into countries through the medium of hides, wool, and hair of animals that have succumbed to this malady. From this it may be inferred that the difficulty in controlling anthrax is due to the many and varied media by which the specific germ may be conveyed; and which, it should be stated, will remain infective in the spore form for years, and then be capable of doing more damage. But, although this disease is so easily and often so widely distributed, it is possible through a knowledge of the life-history of the organism, and by applying those principles of sanitary science already alluded to, both of which are readily obtained in this progressive age, to be able to destroy infection and thereby prevent an outbreak, at least, when we have a first case to deal with.

It has been proven that if all the natural openings in the body of an anthrax victim are stopped up with some material saturated with a good germicidal agent, as carbolic acid, to prevent escape of virulent blood, the germs in the blood of the body will degenerate and die for want of oxygen, without which they cannot exist, and if that carcass is effectually cremated it will be impossible for any of the organisms to have survived such a "warm time." If, then, such measures as these, simple as they may appear, had been adopted in earlier times in that section of the country to which I have referred, anthrax, if not altogether eradicated, would have been very much restricted in its area of infection. Since, however, a more intelligent knowledge of the true cause and the nature of the infection has been acquired, sanitary measures are having a very salutary effect, and although it is a somewhat difficult matter to completely exterminate anthrax infection from lands that have been infected and reinfected for years through the contaminating influence of diseased carcasses left carelessly exposed upon their surface, yet, by a more lively regard for the value of stricter sanitary measures to limit the spread of the disease, combined with a system of preventive inoculation of our animals against it, its devastating effects have been materially reduced during the past few years, which gives hope of perhaps getting rid of the disease eventually in that part of the country.

I hoped to have been able to say a word or two about Texas or Southern cattle fever, but have already occupied too much of the valuable time of the Association. I will take the liberty of stating, however, that this disease which hitherto proved so fatal to Northern cattle coming South, destroying anywhere from 50 to 90 per cent.

of them, and was the dreaded bane and bugbear of the Southern stockman who was desirous of importing animals of pure breeding to improve and build up his degenerated herds, has been at last "shorn of its terrors," to him, at least, by the immunizing method of blood inoculation. On the other hand, the matter of preventing infection being carried north of the fever line by tick-infested cattle of the South is under the control of the Bureau of Animal Industry at Washington, and the work is successfully accomplished through a rigid system of inspection and quarantine laws. The infective agent in this disease belongs to the animal kingdom, like the germ of malaria, and transmitted by means of the cattle-tick, while those responsible for the other diseases mentioned are bacteria and are members of the vegetable kingdom.

Finally, allow me to state that, to be able to intelligently identify and control a disease of a specific or infectious nature involves on the part of the expert two lines of study. First, the study of the effects produced by the infective germ upon the system of the victim, or what we know as symptoms; the second, a study of the organism itself, including its form, life-history, and everything else that is to be known about it in order to control it, as it, too, is a living entity. An accurate knowledge of each or both can only be acquired by years of painstaking, persistent effort and trained observation. Individuals so equipped are to be found among those who are laboring along these lines for the good of the live-stock interests of the country, from the workers connected with that special branch of the National Department of Agriculture downward, and if, by the earnest co-operation of those who have, or should have, the greatest personal interest in the welfare of the industry—the stock-owners themselves—to afford adequate encouragement and assistance to these workers in their laudable endeavors, the infectious diseases of our farm animals would the sooner be "robbed of their sting," and the whole country be the gainer accordingly.

